# Perioperative outcomes following robot-assisted partial nephrectomy for renal cell carcinoma according to surgeon generation

**DOI:** 10.1186/s12893-022-01654-4

**Published:** 2022-05-26

**Authors:** Makoto Toguchi, Tsunenori Kondo, Kazuhiko Yoshida, Kazunari Tanabe, Toshio Takagi

**Affiliations:** 1 Department of Urology, Tokyo Women’s Medical University, 8-1 Kawada-cho, Shinjuku-ku, Tokyo, 162-8666 Japan; 2 Department of Urology, Tokyo Women’s Medical University Medical Center East, Tokyo, Japan

**Keywords:** Kidney neoplasm, Learning curve, Nephrectomy, Outcome, Robotics

## Abstract

**Objective:**

The experience of performing robot-assisted partial nephrectomy (RAPN) is associated with better surgical outcomes. However, surgeon’s generation may impact surgical outcomes. We evaluated the perioperative outcomes of RAPN between first- and second-generation surgeons according to the surgeon’s experience.

**Methods:**

This study included 529 patients who underwent RAPN for renal cell carcinoma from January 2013 to November 2018. Four specific surgeons performed the surgery. According to the surgeon’s generation, the patients were divided into two groups: first-generation and second-generation. To reflect the learning curve of RAPN, the surgical outcomes of each case (1–50, 51–100, 101–150) were evaluated between these groups.

**Results:**

Between 1 to 50 cases and 101–150 cases, no significant differences in patient characteristics were observed between the two generations. Between 51–100 cases, age at surgery was significantly younger in the first-generation than in the second-generation group (58 years vs. 64 years, p = 0.04). The second-generation group had a shorter operation time in cases 1–50 (169 min vs. 188 min, p = 0.0001), 51–100 (145 min vs. 169 min, p = 0.008), and 101–150 (142 min vs. 165 min, p = 0.009), than the first-generation group. Although shorter WIT and higher trifecta achievement were observed in the second-generation group than in the first-generation group between 1–50 cases, the difference was not noted between 51–100 cases and 101–150 cases.

**Conclusion:**

Patients operated by second-generation surgeons had better surgical outcomes than first-generation surgeons, especially during the early experience period, which might result from their assistance experience, sophisticated surgical procedures refined by the first-generation, and the first-generation surgeon’s introduction.

## Introduction

Robot-assisted laparoscopic partial nephrectomy (RAPN) is used for the treatment of small renal masses, and its indication has been gradually expanded for more challenging cases. In fact, 95% of $\le$7 cm renal masses have been removed by RAPN in our institution so far. Perioperative outcomes of RAPN are dependent on tumor factors such as tumor location or size; patient factors such as comorbidities or body mass index (BMI); and the surgeon’s experience. Regarding the surgeons’ experience, several studies have investigated the learning curves for RAPN and demonstrated that an acceptable warm ischemia time (WIT), estimated blood loss (EBL), and operation time could be achieved within 25–30 cases[1, 2], with a plateau reached within 70–75 cases.

However, RAPN is performed by not only surgeons but also assistants. In addition, surgeries are refined, well-prepared, and unwasted procedure which is organized by the surgical team. Aeuschner et al. investigated the impact on surgical outcomes of RAPN according to several experience factors such as the department, the surgeon, and the assistant, and demonstrated that perioperative outcomes improved significantly with experience greater than 100 for the department, experience greater than 35 for the surgeon, and experience greater than 15 for the assistant [3].

The experience of the department includes the primary surgeons or subsequent surgeons who assist primary surgeons as supposed future surgeons. First-generation surgeons are required to establish optimal surgical methods; therefore, their learning curve of RAPN may be slow, while that of second-generation surgeons, who receive support from first-generation surgeons, may be better due to their assistant experience and lack of wasted procedures that were refined by first-generation surgeons. In this study, we assessed the impact on perioperative outcomes of patients who had undergone RAPN between first- and second-generation surgeons according to the surgeon’s experience.

## Patients and methods

### Patient population and study design

This study included 836 patients who underwent RAPN for renal tumors from January 2013 to November 2018 at Tokyo Women’s Medical University Hospital. The patients were divided into two groups, first-generation and second-generation. The first-generation group was defined as two surgeons who introduced RAPN at our institution. Although these two surgeons had little experience with RAPN assistants, they performed more than 100 cases of laparoscopic partial nephrectomy and more than 100 cases of robot-assisted laparoscopic radical prostatectomy before working with RAPN. The second-generation group was defined as another two surgeons who succeeded, as assistants, in the RAPN surgery led by the first-generation surgeons. In addition, the first-generation surgeons assisted or supervised the second-generation surgeons in the initial 10 or 20 RAPNs.The patients who underwent RAPN under surgeons other than the specific four surgeons and those with insufficient medical records were excluded from the study. Eventually, 529 patients were included as subjects of this study.

The Internal Ethics Review Board of Tokyo Women’s Medical University approved our retrospective study (Institutional Review Board approval no. 2020-0062). It was carried out in accordance with the principles outlined in the Declaration of Helsinki. The requirement for written informed consent was waived due to the retrospective nature of the study.

### Data collection

Preoperative demographic factors analyzed included gender, age, body mass index (BMI), preoperative renal function, and American Society of Anesthesiologists classification (ASA) score, tumor size, and RENAL-NS [4]. Perioperative factors analyzed included trifecta achievement rate, total operative time, WIT, surgical margin status, and complications that were recorded using the Clavien classification system [5]. Trifecta was defined as WIT < 25 min, negative surgical margins, and no perioperative complications (Clavien grade $\ge$2). The estimated glomerular filtration rate (eGFR) was calculated using the Modification in Renal Disease 2 equation proposed by the Japanese Society of Nephrology. The equation is as follows:

eGFR = 194 × serum creatinine (mg/dl)^−1.904^ × age^−0.287^ (× 0.739, if female) [6].

### Surgery

The surgical method of RAPN has been described previously [7]. Briefly, surgeries were performed through four robotic arms and one or two assistant ports. The renal hilum was dissected, and the renal artery was identified. Thereafter, surrounding tissue and fat were removed from the tumor margin, and a resection line was determined using ultrasound. The disappearance of renal vessel flow was confirmed by ultrasound after renal artery clamping; the tumor was then resected. The tumor bed was ligated using barbed sutures, and the renal artery was un-clamped. After confirming arterial hemostasis, renorrhaphy was placed using a running suture. The surgical approach, including retroperitoneal and transperitoneal, depended on the tumor position and surgical history [7]. All surgeries were performed under warm ischemia.

### Outcomes

The primary outcome was the comparison of surgical outcomes, including operation time, WIT, EBL, perioperative complications (Clavien grade 2 or more), positive surgical margin rate, postoperative renal function, and trifecta achievement between the first-generation and second-generation surgeons. To reflect the learning curve of RAPN, the surgical outcomes of each case (n = 50) were evaluated between groups.

### Statistical analysis

Statistical analysis was performed using JMP Pro v.15. Comparisons between groups were performed using χ^2^ and student t-tests for categorical and continuous variables, respectively. The data for an operative time as a function of case number was modeled via polynomial regression. A p value < 0.05 was considered statistically significant.

## Results

Baseline demographics, radiographic tumor characteristics, and preoperative variables are summarized in Table 1. The mean patient age was 58 years, and 72% of patients were male. The mean BMI was 24 kg/m^2^, and the distribution of the ASA score was one in 105 patients (10%), two in 385 patients (73%), and three in 39 patients (7%). The number of patients with a medical history of diabetes mellitus (DM) and HTN was 87 (16%) and 227 (43%), respectively. The mean tumor size was 29 mm, and the R.E.N.A.L nephrometry score was low in 206 patients (39%), intermediate in 271 patients (51%), and high in 52 patients (10%).

**Table 1:** Clinical characteristics of 529 consecutive patients

Sex, male, n	382 (72)
Age, yr	58 (48–67)
ASA	
1	105 (20)
2	385 (73)
3	39 (7)
BMI, kg/m.^2^	24 (22–27)
DM, n	87 (16)
HTN, n	227 (43)
Preoperative eGFR, ml/min/1.73 m^2^	67 (58–79)
Tumor size, mm	29 (20–39)
R.E.N.A.L nephrometry score	
Low	206 (39)
Intermediate	271 (51)
High	52 (10)

Values are presented as number (%) or mean (interquartile range)

*ASA* American Anesthesiologists Association, *BMI* body mass index, *DM* diabetes mellitus, *HTN* hypertension, *eGFR* estimated glomerular filtration rate

A comparison of patient characteristics between first- and second-generation surgeons stratified by 50 consecutive cases is shown in Table 2. Between 1 to 50 cases, no significant differences in patient characteristics were observed between the two generations. Between 51–100 cases, the age at surgery was significantly younger in the first-generation than in the second-generation group (58 years vs. 64 years, p = 0.04). Other factors were not significantly different.

**Table 2:** Comparisons of patients' characteristics between first- and second-generation surgeons

Surgeon's experience	1–50			51–100			101–150		
	First	Second	*p*	First	Second	*p*	First	Second	*p*
Number of patients	100	100		98	100		50	81	
Age, yr	60 (51–67)	55 (45–67)	0.141	58 (47–66)	64 (52–70)	0.04	59 (46–67)	59 (46–66)	0.737
BMI, kg/m.^2^	24.1 (22.6–26.1)	23.2 (21.6–26.6)	0.459	23.9 (21.6–26.9)	23.4821.8–26.39	0.255	23 (21.1–26.8)	24.3 (21.7–26.7)	0.819
Preop eGFR, ml/min/1.72 m.^2^	66.3 (58.4–76.1)	68.2 (56.2–80.8)	0.446	70.8 (55.9–78.7)	65.8 (56.4–75.3)	0.503	70.9 (60.1–82.3)	696 (59.1–79.9)	0.38
Tumor size, mm	28 (22–35)	30 (20–41)	0.265	30 (22–38)	28 (18–42)	0.603	30 (21–36)	26 (18–44)	0.486
RENAL-NS			0.0791			0.9809			0.1287
Low (4,5,6)	41 (41)	53 (53)		33 (34)	35 (35)		12 (24)	32 (40)	
Intermediate (7,8,9)	48 (48)	43 (43)		55 (56)	55 (55)		29 (48)	41(51)	
High (10)	11 (11)	4 (4)		10 (10)	10 (10)		9 (18)	8 (10)	
ASA score			0.2190			0.2927			0.9405
1	25 (25)	16 (16)		15 (15)	21 (21)		10 (20)	18 (22)	
2	71 (71)	77 (77)		72 (73)	73 (73)		36 (72)	56 (69)	
3	4 (4)	7 (7)		11 (11)	6 (6)		4 (8)	7 (9)	

Values are presented as number (%) or median (interquartile range)

*ASA* American Society of Anesthesiologist, *BMI* body mass index, *preop* preoperative, *NS* nephrometry score, *eGFR* estimated glomerular filtration rate

The comparisons of surgical outcomes between first- and second-generation surgeons are shown in Table 3. Between 1 and 50 cases, a shorter operation time (169 min vs. 188 min, p = 0.0001) was observed in the second-generation group than in the first-generation group, and this trend was also observed in 51 to 100 cases (145 min vs. 169 min, p = 0.008) and 101 to 150 cases (142 min vs. 165 min, p = 0.0009). In addition, shorter WIT was observed in the second-generation group compared to the first-generation group between 1 to 50 cases (17 min vs. 20 min, p = 0.019); however, no significance was observed in 51 to 100 cases (p = 0.812) and 101 to 150 cases (p = 0.489). Other surgical outcomes, including EBL, perioperative complications, positive surgical margin rate, and postoperative renal function, were not significantly different between the two groups over three surgeons’ experience. Trifecta achievement rate was significantly higher in the second-generation group than in the first-generation group (81% vs. 58%, p = 0.0004) between 1 and 50 cases. However, no significance was observed in 51 to 100 cases (72 vs. 66%, p = 0.387) and in 101 to 150 cases (78 vs. 80%, p = 0.763).

**Table 3:** Comparisons of surgical outcomes between first- and second-generation surgeons

Surgeon's experience	1 to 50			51–100			101–150		
	First	second	*p*	First	Second	*p*	First	Second	*p*
Operation time, min	188 (165–213)	169 (147–190)	0.0001	169 (139–197)	145 (127–178)	0.008	165 (155–186)	142 (126–178)	0.009
WIT, min	20 (14–26)	17 (14–20)	0.019	17 (13–24)	17 (12–24)	0.812	17 (12–19)	15 (12–21)	0.489
EBL, ml	30 (10–100)	50 (20–100)	0.174	30 (10–100)	30 (10–50)	0.142	18 (10–42)	50 (14–90)	0.081
Clavien $\ge$ 2, n	21 (21)	14 (14)	0.193	18 (18)	10 (10)	0.091	10 (20)	7 (9)	0.06
Positive margin, n	0	0	–	1 (1)	0	0.311	0	4 (5)	0.111
Postoperative eGFR, (1 month after), ml/min/1.72m2	60.5 (51.3–70.1)	63.9 (53.9–74.8)	0.198	64.4 (48.3–73.9)	60.7 (49.9–73.2)	0.925	67.2 (53.5–76.1)	65.8 (52.9–78.7)	0.526
Trifecta, n	58(58)	81(81)	0.0004	65(66)	72(72)	0.387	40(80)	63(78)	0.763

Values are presented as number (%) or median (Interquartile range)

*ASA* American Society of Anesthesiologists, *BMI* body mass index, *preop* preoperative, *NS* nephrometry score, *eGFR* estimated glomerular filtration rate, *EBL* estimate blood loss

**Fig. 1:**
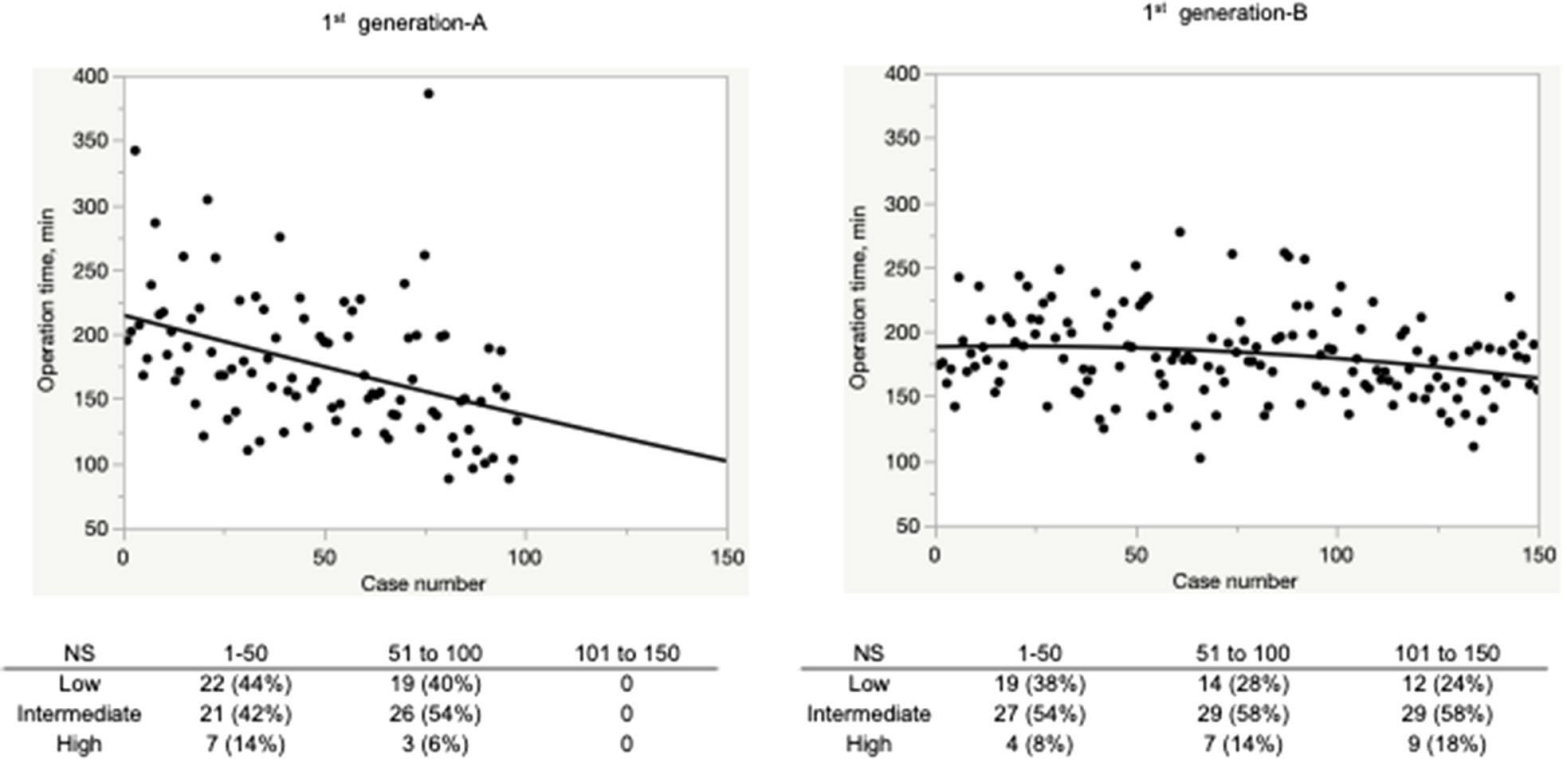
The learning curve of operation time in first-generation surgeons. A steep slope reduction and moderate slope reduction were shown in 1st generation-A and 1st generation-B, respectively

**Fig. 2:**
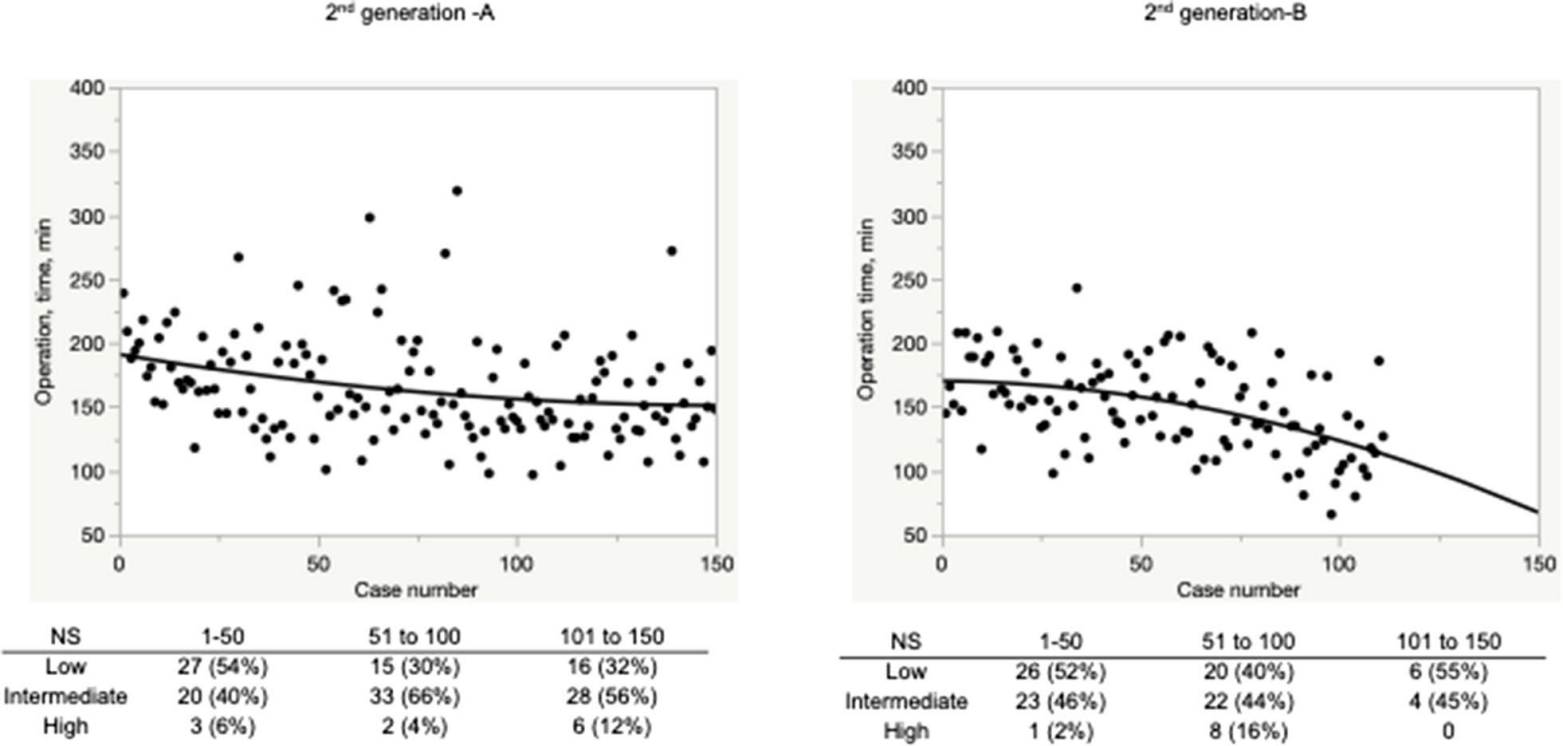
The learning curve of operation time in second-generation surgeons. A moderate slope reduction was observed in 2nd generation-A and 2nd generation-B

Figures 1 and 2 showed the learning curve of operation time in the first-generation group and second-generation group, respectively. A steep slope reduction was observed in Fig. 1A, even though there was a decreased number of highly complex tumors. The moderate slope reduction was observed in Figs. 1B, 2A, B, despite an increased number of complex tumors.

## Discussion

In this study, we investigated the surgical outcomes of RAPN according to surgeons’ experience. Even in a high-volume center, one or two specific surgeons (first-generation) commence new surgeries and improve their procedures, and then, subsequent surgeons (second-generation) turn over the surgeries. Second-generation surgeons usually participate in first-generation surgeons’ surgery as an assistant, and they start their surgery according to refined procedures and techniques developed by first-generation surgeons. Therefore, we hypothesized that second-generation surgeons could achieve better surgical outcomes in their early experience period when compared to first-generation surgeons. As shown in our results, we demonstrated that second-generation surgeons tended to be associated with better surgical outcomes, including shorter operation time and better trifecta achievement rate than first-generation surgeons, in the early experience period. This result suggests the importance of an institutional training system to shorten the period of expertise for RAPN.

The importance of institutional experience in RAPN was shown by Zeuschner et al. [3]. They compared the impact of the learning curve of the department, the console surgeon, the bedside assistant, and patient-related factors on the perioperative outcomes of RAPN. The console surgeon’s experience significantly impacted operation time, EBL, complication rate, and length of hospital stay. In contrast, the experience of the department and bedside assistant were significantly associated with more favorable outcomes in terms of the operation time and open conversion rate [3]. Dagenais et al. evaluated the variability in PN outcomes by physician-level discrepancies. They demonstrated that a high proportion of surgeon factors were associated with the length of hospital stay (90%), positive margins (100%), complications (100%), and 30-readmission (90%) in terms of between-surgeon variability. In contrast, a small to moderate proportion of surgeon factor in operative time (20%), estimated blood loss (40%), ischemia time (10%), and excisional volume loss (18%) were observed. As to operative time, unexplained surgeon factors (27%) and unexplained patients factors (54%) were associated with between-surgeon variability, which may include institutional experience or bedside assistance [8]. According to these previous articles, our results of better surgical outcomes in the second-generation surgeons than in first-generation surgeons may have resulted from the accumulation of skills among all participants, including operation staff.

The evaluation of the learning curve for robotic surgery was assessed by several approaches. Meier et al. evaluated the number of repetitions required to reach the expert level using the da Vinci Surgical Skills Simulator™. They showed that robotic surgeons, table-side assistants, and novice surgeons aged 25 years or younger achieved better results than laparoscopic and open surgeons who had no robotic surgery experience and the older novice group [9, 10]. From this study, the importance of the experience of robotic surgery for robotic skills was demonstrated, whether or not a primary surgeon was shown [9, 10].

On the other hand, laparoscopic experience improves the learning curve in real-world robotic RAPN. Pieroprazio et al. examined the transition to RAPN from pure laparoscopic partial nephrectomy (LPN) and investigated the learning curve; they demonstrated that after a learning experience of approximately 25 cases, the transition from LPN to RAPN can be performed without an additional learning curve and can be associated with immediate benefits [2].

Besides urological robotic surgeries, Pernar et al. reviewed the literature on the learning curve in robotic general surgery [11]. Although there are several outcomes of robotic surgeries, time was used to measure the learning curve in all studies. The learning curve of general surgery has focused on the time under robot support; the number of operations required until acquiring surgical proficiency is increasing. The number of cases needed to achieve plateau performance was wide-ranging but overlapping for different kinds of operations: 19–128 cases for colorectal, 8–95 for foregut/bariatric, 20–48 for biliary, and 10–80 for solid organ surgery [11]. Regarding RAPN, WIT and console time tended to be a measure of the learning curve. Mottrie et al. evaluated the impact of the learning curve on perioperative outcomes in patients who underwent RAPN and demonstrated that WIT (< 20 min) and console times were optimized after the first 30 (p < 0.001) and 20 cases (p < 0.001), respectively[12]. In addition, Larcher et al. performed a similar study and demonstrated that WIT showed a steep slope reduction within the first 100 cases, and a plateau was then observed after 150 cases [13].

Regarding our study, although the steep slope reduction of operation time was observed in Fig. 1A, a moderate slope reduction was shown in Figs. 1B, 2A, B, which might be caused by the increasing number of challenging cases with increasing experience.

The learning curve of surgical outcomes other than time has been described in several studies. Mottrie et al. reported that the complication rates remained unchanged over the entire series, concluding that the learning curve for RAPN is short [12]. On the other hand, Larcher et al. described a linear relationship between experience and complication-free course, which did not reach a plateau, even after 300 cases, concluding that the learning curve appears endless with respect to complications [13]. The two studies contained different cohort sizes and different total complication rates; therefore, apparent controversial results might be influenced by several background characteristics.

The present study had several limitations that should be noted. First, the retrospective nature with data collected from a single institution and a population of tertiary care patients are limitations. Second, four surgeons who performed RAPN in this study had adequate laparoscopic experience, which led to relatively good surgical outcomes in their early period of robotic surgery. Given that surgeons without sufficient experience in laparoscopic kidney surgery were included, the results might have been different. Third, comparisons of surgical outcomes between first-generation and second-generation surgeons were performed with univariate analysis, even though there were similarities in patients and tumor background between the two groups. The strength of our study is that it is a relatively rare study investigating the surgical outcomes between surgeons’ generation. In the early period of experience, shorter operation time and higher trifecta achievement were observed with second-generation surgeons, which could stress the importance of an institutional training system.

## Conclusion

This study showed more favorable surgical outcomes, including shorter operation time and a higher achievement rate in second-generation surgeons than in first-generation surgeons, especially in the early period of their experiences. The second-generation surgeons participated in the surgeries performed by first-generation surgeons as assistants and received support from first-generation surgeons when they started their surgeries as primary surgeons, which may explain our results. Our study suggests the importance of an institutional training system to achieve better surgical outcomes in the early experience period for next-generation surgeons.

## Data Availability

The datasets generated during and/or analyzed the current study are available from the correspondence author on reasonable request.

## Abbreviations

RAPN: Robot-assisted laparoscopic partial nephrectomy
eGFR: Estimated glomerular filtration rate
BMI: Body mass index
WIT: Warm ischemia time
EBL: Estimated blood loss
DM: Diabetes mellitus
ASA: American Society of Anesthesiologists
eGFR: Estimated glomerular filtration rate
HTN: Hypertension
